# District nurses’ experiences of using a clinical decision support system and an assessment tool at elderly care units in primary health care: a qualitative study

**DOI:** 10.1017/S1463423621000530

**Published:** 2021-09-15

**Authors:** Annica Lagerin, Lena Törnkvist, Johan Fastbom, Lena Lundh

**Affiliations:** 1 Department of Health Care Sciences, Ersta Sköndal Bräcke University College, Stockholm, Sweden; 2 Division of Family Medicine and Primary Care, Department of Neurobiology, Care Sciences and Society, Karolinska Institutet, Stockholm, Sweden; 3 Head of Continuing Professional Education and Health Care Development Unit, Academic Primary Health Care Centre, Region Stockholm, Sweden; 4 Aging Research Center (ARC), Karolinska Institutet Stockholm University, Stockholm, Sweden

**Keywords:** clinical decision support system, district nurse, medication, older adult, primary health care, tool

## Abstract

**Aim:**

The present study aimed to describe the experience of district nurses (DNs) in using a clinical decision support system (CDSS) and the safe medication assessment (SMA) tool during patient visits to elderly care units at primary health care centres.

**Background:**

In Swedish primary health care, general practitioners (GPs) prescribe and have the responsibility to regularly review older adults’ medications, while DN (nurses specialised in primary health care) play an important role in assessing older adults’ ability to manage their medications, detecting potential drug-related problems and communicating with patients and GPs about such problems. In a previous feasibility study, we found that DNs who use a combination of a CDSS and the SMA tool identified numerous potentially harmful or dangerous factors and took a number of nursing care actions to improve the safety and quality of patients’ medication use. In telephone interviews, patients indicated that they were positive towards the assessment and interventions.

**Methods:**

Individual interviews with seven DNs who worked at six different primary health care centres in Region Stockholm were carried out in 2018. In 2019, an additional group interview was conducted with two of the seven DNs so they could discuss and comment on preliminary findings. Qualitative content analysis was used to analyse the interview transcripts.

*Findings:* Using the tools, the DNs could have a *natural conversation* about medication use with older adults. They could get a *clear picture* of the older adults’ medication use and thus obtain information that could facilitate *collaboration* with GPs about this important component of health care for older adults. However, for the tools to be used in clinical practice, some barriers would have to be overcome, such as the time-consuming nature of using the tools and the lack of established routines for interprofessional collaboration regarding medication discussions.

## Introduction

During the past several decades, medication use has increased more rapidly in older people than in other groups (Hovstadius *et al.*, 2010), mainly because of the introduction of new drugs and treatment guidelines (Morin *et al.*, 2015; Lagerin *et al.*, 2017). At the same time, age-related physiological changes in drug turnover and sensitivity to drugs increase the risk of adverse drug reactions (ADRs) (Bergqvist *et al.*, 2009; Kim Jennifer, 2017), which is further augmented by the use of multiple drugs (polypharmacy). Nearly half of those aged 75 years and older in Sweden are exposed to polypharmacy (Wastesson *et al.*, 2018), which increases the risk of ADR and drug–drug interactions and has a negative impact on medication adherence (Hughes, 2004; Lagerin *et al.*, 2014; Kim, 2017).

As in most western countries, the majority of treatment for older adults in Sweden is provided by primary health care, and drugs are the main medical treatment (Wastesson *et al.*, 2018). In the Swedish primary health care system, both general practitioners (GPs) and district nurses (DNs) work with older patients’ medications. GPs are responsible for prescribing and regularly reviewing medications, while DNs play an important role in assessing older patients’ ability to manage their medications, detecting potential drug-related problems and communicating with patients and GPs about such problems (Lagerin *et al.*, 2014). DNs are specialist nurses whose main responsibilities include preventing illness in the population and planning, providing and evaluating care (Lagerin *et al.*, 2014). They typically work at primary health care centres, collaborating with GPs and other health care professionals (Sherman *et al.*, 2016) but can also work in the community, collaborating with municipal personnel (e.g., home help services).

Since the beginning of the 2000s, there have been significant improvements in older adults’ medication use in Sweden (Morin *et al.*, 2015). These include reduced use of inappropriate drugs, inappropriate drug combinations and some psychotropic drugs. In contrast, the use of various somatic drug treatments (e.g., cardiovascular and anticoagulant medications) has increased steadily, and with it, overall drug use. As a consequence, medication use in older adults has become increasingly complex, and more regular reassessments and refinements will be required to maintain an adequate risk–benefit balance.

In 2016, the Executive Board of the Stockholm County Council (now Region Stockholm) decided to offer each primary health care centre the opportunity to establish an elderly care unit at the centre. The purpose of the unit is to provide support and security for older adults by increasing the availability and continuity of care for patients 75 years and older. Two of the elderly care units’ tasks are performing medication reviews and developing care plans for older adults.

In a previous feasibility study, we investigated whether DNs can use a combination of a clinical decision support system (CDSS) and an assessment tool to help improve medication use in patients who visit elderly care units (Lagerin *et al.*, 2020). The CDSS is a web-based, patient-centred tool for analysing the quality of older adults’ medication use (Björkman I, 2012). It can be used either by the patient at home or by physicians or nurses together with the patient. The CDSS provides information about current drug use and symptoms that are potentially drug-related, as well as an analysis of the quality of drug use and potential ADRs (Björkman I, 2012). The analysis generates a printable “basis for discussion” intended to empower the patient and stimulate the dialog with the physician or nurse. The printout also allows the health care professional to study the patient’s current list of drugs and to assess any reported symptoms. The second tool is the 20-item safe medication assessment (SMA) tool, which DNs are to use for assessing and identifying potential problems with a patient’s medication management (Gusdal *et al.*, 2011) and for promoting safe medication use (Lagerin *et al.*, 2014).

In the feasibility study, nine DNs from seven primary health care centres used the CDSS and SMA with 45 patients between 2017 and 2018. The DNs, all of whom were women, identified numerous potentially harmful or dangerous factors and took a number of nursing care actions to improve the quality and safety of patients’ medication use. In telephone interviews, patients indicated that they were positive towards the assessment and interventions. The aim of the present study was to describe the DNs’ experiences of using the CDSS and the SMA tool during patient visits to elderly care units at primary health care centres.

## Methods

### Study design and sample

The design was qualitative and descriptive. In 2018, seven individual interviews were conducted with DNs. Individual interviews were chosen because they give participants the opportunity to provide detailed information about the topic under study (Kvale and Brinkmann, 2014). In 2019, an additional group interview was conducted with two of the seven DNs so that they could discuss and comment on the preliminary findings. The group interview format was chosen to stimulate discussion and generate information that otherwise might not emerge (Kvale and Brinkmann, 2014). We chose to analyse the interview transcripts with qualitative content analysis (Graneheim *et al.*, 2017) because it is an appropriate method for exploring and describing variation in experiences(Graneheim *et al.*, 2017).

### Participants and setting

In 2018, all nine DNs who participated in the original feasibility study (Lagerin *et al.*, 2020) were invited for an individual interview and seven of these agreed to take part in the present study. They had all completed an initial two-day course on older adults’ medication use and polypharmacy. The course also covered how to use the CDSS and SMA tool. The DNs worked at six different primary health care centres in Stockholm County, Sweden, and were all active at their centre’s elderly care unit. Their mean age was 57 years (range 42–65 years). One was training to become a DN. The others had worked as DNs for an average of 19 years (range 4–33 years) (Lagerin *et al.*, 2020).

### Interviews

The first author (AL) interviewed the seven DNs individually at their own workplace when she travelled there to pick up the paperwork from the completed feasibility study. The interviews were guided by a semi-structured list of mainly open-ended questions. The same questions were asked about the CDSS and SMA: 1) What is your overall impression of using the tools during patient visits at an elderly care unit? What worked well? What worked less well? Was there anything missing? 2) How much time did you spend discussing the patients’ drug treatment? 3) Would you like to use the tools during additional patient visits? Follow-up questions were asked to probe any new information that came up during the dialogue. Interviews lasted for approximately 30 min (range 20–40 min), were audio recorded and transcribed verbatim. The transcript of each interview was coded with a number for each DN. The code after each quotation refers to each extract (DN 1, DN 2, DN 3, etc.).

In 2019, after the preliminary interview analyses were completed, AL invited all seven DNs to an additional group interview at a college in Stockholm. Only two participated in this interview. One declined to participate because she had retired, one explained that she could not get time off, one chose not to participate because she had changed jobs and two did not provide a reason. At the interview, AL presented the three main categories and their subcategories, divided into barriers and facilitators (Table 1) and invited the DNs to discuss and comment on these findings. AL facilitated the discussion and LL acted as observer. LL took notes during the interview and summarised the discussions at the end of the interview, which took 55 min, was audio recorded and transcribed verbatim.

**Table 1.** Illustration of the process of the content analysis

Meaning unit	Condensed meaning unit	Code	**Subcategory (** ***label*** **)**	Main category
“Yes, mostly hearing … you had to repeat the questions many times so they didn’t misunderstand and ask follow-up questions, so it takes time”	Problems with hearing and a need to repeat questions to avoid misunderstanding	Problems with hearing	Older adult had vision or hearing loss (*barrier*)	Natural conversation
“Because I often booked a doctor’s appointment for them and made sure everything was prepared”	Book GP visit for follow-up	GP visit	DNs gained information that could form basis for nursing care decisions (*facilitator*)	Clear picture
“Of course you didn’t get feedback from all the doctors”	Lack of feedback from doctors	Feedback	Primary health care centres lacked established routines for interprofessional collaboration regarding medication discussions (*barrier*)	Collaboration

### Data analysis

Data analysis began with reading the transcripts several times to obtain an overall picture of the DNs’ experiences of using the tools during patient visits (Graneheim *et al.*, 2017). Interview texts relevant to the study aim were then divided into meaning units, which were condensed, abstracted and labelled with a code that reflected the manifest content (Table 1). The codes were sorted into subcategories, which were labelled as either barriers or facilitators. The subcategories – both barriers and facilitators – were then sorted into three main categories: *natural conversation*, *clear picture* and *collaboration*. When the researchers found it necessary, subcategories and categories were moved between main categories to ensure meaningfulness and coherence. AL and LL conducted separate analyses and then discussed their findings with each other and with LT until they reached consensus.

## Results

DNs were eager to achieve a *natural conversation* with the older adults about their medication use, a *clear picture* of medication use and *collaboration* with the older adults’ GP regarding medication use. They experienced facilitators of and barriers to achieving these goals. Some facilitators and barriers were related to the tools, whereas others were related to the DNs, the older adults or the primary health care centre (Table 2). In the following section, the results are illustrated with quotations from the interviews. Omissions of less than a full sentence are indicated with three dots and omissions of one or more sentences with four dots. Square brackets indicate clarifying words added by the authors.

**Table 2.** District nurses’ experiences of barriers to and facilitators to achieving a natural conversation, gaining a clear picture and collaboration with GPs when using the clinical decisions support system and safe medication assessment tool during patient visits to elderly care units

Main category	Subcategories	
	Facilitators	Barriers
Natural conversation	*Older adult involved in medication discussion*	*Time consuming to use the tools*
	*Older adult gained a feeling of security*	*Older adult could become tired*
	*Older adult appreciated the opportunity for a medication conversation*	*Older adult felt it was demanding to respond to the questions*
	*Older adult was interested in technology*	*Older adult had vision or hearing loss*
	*Older adult wanted to tell the DN about their medications*	*Older adult sat quietly*
	*Older adult had prepared for the conversation*	
Clear picture	*DNs gained an overview of the older adult’s medication use*	*DNs feared causing anxiety*
	*DNs gained information and knowledge about the older adults’ medication use*	*Older adult lacked knowledge about his or her medications*
	*DNs gained information that could form basis for nursing care decisions*	*Technical problems with the CDSS*
		*Not enough space on the SMA tool to fill in the information gathered*
Collaboration	*DNs gained information that could form the basis for discussion with GPs*	*Primary health care centres lacked established routines for interprofessional collaboration regarding medication discussions*
	*DNs gained a feeling of security in their professional role*	*GPs did not seem interested in the information*
		*Older adults did not have their own regular GP*

CDSS, clinical decision support system; DN, district nurse; GP, general practitioner; SMA, safe medication assessment tool.

### Natural conversation

According to the DNs, a natural conversation about medication use meant that the conversation was easy and open, which created the opportunity to obtain information.

I asked the patient to tell me, and then I also produced the list of medications, and sometimes it was different. Sometimes the medications the patients mention are on the list, and then I asked how that was, and then it turns out that the patient may not take it anymore. Or it’s the other way around – sometimes the patient may have bought some extra medication …. It could be omeprazole or something for constipation that they bought themselves at the pharmacy and that they didn’t have a prescription for. (DN 7)

#### Factors that facilitated a natural conversation

The questions in the tools helped ensure that the *older adults were involved in the medication discussion*, which facilitated a natural conversation. According to the DNs, many older adults in the study felt specially selected and noticed and thought that their views were taken into account during the conversation. One DN said, “I think the older adults felt seen and noticed and that their views were perhaps taken a little more seriously” (DN 6).

DNs also thought that the *older adults gained a feeling of security* via the analyses. Both the situation surrounding the discussion (enough time, reasonable tempo) and the questions made the older adults feel calm and safe. Afterwards, many wanted to take a printout of the results of the analysis to their next doctor’s appointment. It also facilitated the discussion if the *older adult appreciated the opportunity for a medication conversation*. According to DNs, many older adults thought it was valuable to be able to go through their medicines with the DN, to be able to talk about their health problems and to have time set aside for this process. It also facilitated the medication discussion if the *older adult was interested in technology*. Many found technology exciting and were positive about using a CDSS. It was also helpful if the *older adult wanted to tell the DN about their medications* and other products that they may have bought from a pharmacy or health food store, as well as about any problems they were experiencing that might be related to these products. The DNs also perceived it as helpful if the *older adult had prepared for the conversation*. This could involve bringing their prescription list from the pharmacy or having read about when and how they should take each medication.

#### Barriers to a natural conversation

DNs explained that it could be *time consuming to use the tools*. According to the DNs, it typically took about an hour to go through the questions. However, an hour was not always enough time, for example, if the older adult had prescriptions for many different medications or experienced side effects or problems that the DN or the older adult wanted to discuss. *Older adults could become tired* because of the length of the review, because they were older (i.e., the oldest old) or because they did not know very much about their medications. DNs said that sometimes they needed to break the visit into two parts or arrange a return visit. “It takes a long time to do, and the patient gets tired after a while if there are many drugs and if they’re a little older”, said DN 1.

A natural conversation could also be more difficult if the *older adult felt it was demanding to respond to the questions* in the tools. According to the DNs, the older adults could feel as if their knowledge was being checked or that their need for medication was being questioned, for example, if they used sleeping pills or painkillers and knew that they were using them inappropriately. It could also be difficult to achieve a natural conversation if the *older adult had vision or hearing loss*. DNs might then need to repeat questions, which made it difficult to have an easy and open two-way discussion. Another barrier occurred if the *older adult sat quietly*. This could happen if the DN was highly focused on the CDSS or was not used to using a CDSS, or if the older adult did not know much about his or her medication and therefore had trouble responding to the questions on the two tools.

### A clear picture

According to the DNs, obtaining a clear picture meant that they gained a good overall grasp of the older adult’s medication use. If the older adult did not understand a question, sometimes the DN could still obtain information by rewording the question somewhat.

You think about the questions, and the questions deepened the conversation, and you put them in different ways. Because sometimes I found that the patient might not understand the question, and then you had to ask the question from a different perspective so that you captured the same – what should I say – meaning, and it became a different discussion. (DN 4)

#### Factors that facilitated a clear picture

The questions in the tools helped the *DNs gain an overview of the older adult’s medication use*. The CDSS and the SMA tool structured the conversation and systematised information gathering. DNs learned, for example, that older adults did not always take medication as prescribed (e.g. diuretics) and that they sometimes thought they were taking too many medications, used medications that were no longer prescribed or took a double dose of a drug.

I had a patient today, for example, who had received two different blood pressure medications of different brands, and then the patient thinks that you should take one tablet in the morning and one tablet in the evening. (DN 4)

The conversation also helped *DNs gain information and knowledge about the older adults’ medication use*. DNs described obtaining information that they did not previously have and could now discuss with the patients, such as learning that the patient had incontinence or difficulty swallowing pills. Furthermore, by using the tools, some DNs learned more than they had known before, for example, about risky medication use. Via the questions, the DNs also *gained information that could form a basis for nursing care decisions*. For example, the DNs could decide to provide self-care advice (e.g. for sleep problems) or could book a medication review appointment with the older adult’s GP.

#### Barriers to gaining a clear picture

One barrier to gaining a clear picture occurred when the *DNs feared causing anxiety* in the older adults. DNs could feel concerned that asking the questions from the tools (for example, asking about a newly prescribed cancer drug) might make an older adult feel anxious. The DNs did not always know how familiar older adults were with certain diseases and/or drug treatments and explained that they did not always feel comfortable bringing up such a serious health problem, especially if they did not know the patient. They could therefore sometimes skip questions on the tools. If DNs noticed during the conversation that the older adult seemed uneasy, they tried to create calm and set aside extra time to talk about the person’s experience of living with a serious disease.

I could sometimes feel afraid of creating anxiety. When they come up with a medication that they have for cancer, and then you wonder like how well they understand the situation and what information they’ve received and so on. (DN 6)

Other situations that made it difficult to gain a clear picture occurred when the *older adult lacked knowledge about his or her medications*, such as not knowing their names. *Technical problems with the CDSS* could also be a barrier. For example, such problems could mean that DNs could not enter the names of the patient’s drugs in the CDSS and/or that the program could not analyse the older adult’s medication use. Additionally, sometimes there was *not enough space on the SMA tool to fill in the information gathered* about the patient’s medications. Some older adults are treated with many medications, sometimes ten or more, and in those cases, the SMA did not have enough space to record the information.

### Collaboration

Collaboration meant that DNs could communicate well with the patient’s GP about possible problems with medication use.

#### Factors that facilitated collaboration about older adults’ medication use

By using the tools, *DNs gained information that could form the basis for discussion with GPs* about older adults’ medication use. By giving the DNs a clearer picture and more information about the patient’s problems and needs, the tools helped clarify the DNs’ work. This, in turn, helped the *DNs gain a feeling of security in their professional role*.

I think it’s part of our role and our own security as district nurses that we somehow become clearer and make an assessment based on how the patient experiences things and how they feel, and whether it’s the medication that has caused this or not. (DN 4)

#### Barriers to collaboration about older adults’ medication use

DNs described how many *primary health care centres lacked established routines for interprofessional collaboration regarding medication discussions*, which made it harder to collaborate with GPs about the results of the discussion. For example, when DNs suspected that the older adult was experiencing a side effect of a medication, it could be difficult to get feedback from the GP. DNs could sometimes find that *GPs did not seem interested in the information* the DNs wanted to discuss (that is, the information that came from the CDSS and/or the SMA tool). They said that some GPs wanted to use their own routines for medication reviews, which did not include collaboration with DNs.

You want the information to go to the doctor. I see the doctors doing their [own] drug reviews. They think it’s great that you do that with SMA, but I [the doctor] do my [own] drug reviews. (DN 1)

The DNs noted that some younger GPs were more involved and wanted to discuss older adults’ drug use on the basis of the analyses from the two tools. Furthermore, at many primary health care centres, the *older adults did not have their own regular GP*, but rather met whichever doctor was available for an appointment. This also made it harder for the DN to follow-up the patients’ medication use.

## Discussion

The findings show that in the DNs’ view, using the CDSS and the SMA tool could lead to outcomes that have the potential to improve care for older adults who attend elderly care units. Using the tools, the DNs could have a natural conversation about medication use with older adults. They could get a clear picture of the older adults’ medication use and thus obtain information that could facilitate collaboration with GPs about this important component of health care for older adults. The findings also suggest that for the tools to lead to these positive outcomes, a number of barriers would have to be overcome. One example is the time-consuming nature of the tools, which means that training and practice are required before they can be used comfortably and efficiently. Another is the need for greater organisational support (support at the primary health care centre) to promote interprofessional collaboration. Many of these barriers could be reduced or eliminated by revising the way the tools are implemented in primary care, including the way the tools are introduced at the primary health care centre and how professionals are trained in using the tools.

### DNs’ views of facilitators

Achieving “a natural conversation” about medication use with an older adult meant involving the older adult in his or her own care. For example, the person could get involved in the medication discussion and/or want to tell the DN about their medications. Studies have found that patients who are involved in their own care rate care quality higher (Slatore *et al.*, 2010) and are more satisfied with care (Dwamena *et al.*, 2012). Involvement in their own care can also help older adults feel safe, especially older adults with chronic conditions (Holmqvist *et al.*, 2019).

According to the DNs, older adults were interested in and appreciated having conversations with a DN about their medication use. Many of them had taken the time and effort to prepare for their elderly care unit visit, and the DNs thought that such preparation facilitated a natural conversation. Furthermore, some older adults took the printout from the CDSS to their next doctor’s visit so that they could continue their medication discussion, this time with their GP. The older adults also indicated that they would be interested in having another medication conversation in the future. These findings are in keeping with those of earlier research showing that older patients want to discuss the safe use of prescribed drugs and to know about the side effects of newly prescribed drugs (Tarn *et al.*, 2015).

The CDSS and the SMA tool helped the DNs obtain a clear picture of the older adults’ medication use. The questions on the tools facilitated conversations about medication by making them systematic and structured. In addition, they enabled DNs to gather new information about the older adults’ health problems. These findings echo those of an earlier study of the SMA (Gusdal *et al.*, 2011). That study found that DNs perceived it as a useful assessment tool, as it alerted them to patients’ attitudes about their medication use and empowered the DNs to identify patients whose medication use was unsafe. In the current study, the DNs carried out several different nursing interventions after using the tools – for example, if the patient reported sleeping problems. This finding is consistent with those of a Norwegian study of barriers to and facilitators of nurses’ use of a CDSS when treating pressure ulcers and malnutrition in older patients. The nurses in that study felt that the CDSS contributed to more professional care and reported that the number of relevant interventions increased after using it (Fossum *et al.*, 2011).

The CDSS and the SMA tool could facilitate DNs’ collaboration with GPs. By providing the DNs with information on what patients needed, the tools could help give the DNs a feeling of security in their professional role as well as information that could form the basis for discussion with GPs. Other studies have found that experienced nurses and physicians may utilise CDSSs in innovative ways, for example, to support their rationale when making decisions (Weber *et al.*, 2009) or as a safety net to check their own clinical judgement (Dowding *et al.*, 2009).

## DNs’ views of barriers when using the tools

DNs found it particularly time consuming to use the tools with the many older adults in the study who used multiple medications (i.e., 10–12 different medications). These older adults almost certainly had multimorbidity. Earlier studies have found, as we did, that time limitations for health care visits make it particularly challenging to optimise medication management in patients with multimorbidity (Fossum *et al.*, 2011; Holmqvist *et al.*, 2019).

DNs could find it difficult to bring up newly prescribed cancer medication. They explained that not knowing the patients well contributed to their discomfort. At the time of the study, elderly care units were relatively new and did not emphasise continuity of care. Care continuity is an important factor in the management of patients with complex health care conditions (Hofer and McDonald, 2019), including multimorbidity (Holmqvist *et al.*, 2019). Better care continuity at the elderly care units would have made DNs more familiar with the patients and perhaps more comfortable with bringing up sensitive drug issues, such as cancer medications. Nurse education may also play a role. GPs are often trained in patient-centred communication, a crucial component of patient-centred care (Altin and Stock, 2016), and medical residents specialising in family medicine are routinely required to learn communication skills (Nasca *et al.*, 2012). However, communication is not among the skills for which credits or learning outcomes are regulated in Swedish nursing programmes (Bullington *et al.*, 2019). Similarly, outside Sweden, few programmes train nurses in how to communicate with patients about difficult topics (Bullington *et al.*, 2019). DNs at elderly care units might be good candidates for continuing professional education in patient-centred communication techniques, especially given the importance of patient-centred, holistic care for patients with multimorbidity (Moffat and Mercer, 2015). Such training would be in keeping with DNs’ responsibility to provide support for people of all ages and medical conditions with a holistic and health-promoting approach (Lagerin *et al.*, 2014).

According to the DNs, barriers to using the tools included a lack of established routines for interprofessional collaboration regarding medication discussion and review. DNs perceived that some GPs wanted to use their own routines when reviewing older patients’ medication lists and that these routines did not involve collaboration with DNs. Our study’s design and implementation may be at least partly responsible for these findings. The CDSS and SMA intervention was approved by each primary health care centre manager, and DNs attended training. However, GPs did not attend the training sessions or participate in the intervention, and we do not know how much information they received about the intervention from their managers. Perhaps interprofessional collaboration would have been better if GPs had been more clearly involved and the intervention had included routines for interprofessional communication about the results. Previous studies provide some support for this idea, as organisational factors that may influence the use of CDSSs include training programmes, social norms, policies and employee empowerment (Powell-Cope *et al.*, 2008). Moreover, a previous study in intensive care units, where both nurses and physicians used a CDSS, found that reports generated by the CDSS facilitated communication between nurses and physicians (Weber *et al.*, 2009).

### Methodological considerations

The researchers involved in this study considered qualitative interviews to be the appropriate method of gathering data to achieve the aim of this study. Relatively few DNs decided to participate, but those who did had a wide variety of experiences of using the CDSS and SMA tool. The first author has professional experience as a DN, which facilitated data collection but could potentially lead to bias. To compensate for this, the first author collaborated closely with the other authors in the analysis of data, discussing the finding until they reached agreement. All the authors who participated in data analysis had also worked as DNs, although not in the past 15 years. Quotations were used to elucidate how the findings were grounded in the data and to strengthen trustworthiness

### Conclusion

The CDSS and the SMA can help nurses gain an overview of patients’ medications, even when patients have many medications. Using the tools can therefore lead to nursing care interventions and collaboration with GPs to improve medication management. Moreover, according to participating DNs, using the tools can help older adults feel seen and involved in their own care and strengthen nurses’ professional confidence. However, for the tools to be used in clinical practice, some barriers would have to be overcome, such as the time-consuming nature of using the tools and the lack of established routines for interprofessional collaboration regarding medication discussions. Many of these barriers could be reduced or eliminated by revising the way the tools are implemented in primary care, including the way they are introduced at primary health care centres and how professionals are trained in using them.
